# Case report: delayed response after electroconvulsive therapy in a patient with major depressive disorder

**DOI:** 10.1186/s12888-021-03053-0

**Published:** 2021-01-21

**Authors:** Fangyue Chen, Emad Sidhom, Sharon Yang, Eladia Ruiz-Mendoza, Julius Essem

**Affiliations:** 1 Peterborough City Hospital, Bretton Gate, Peterborough, UK; 2 Department of Clinical Neurosciences, University of Cambridge, Cambridge, UK; 3 Older People’s Mental Health, Cavell Centre, Edith Cavell Hospital, Peterborough, UK

**Keywords:** Electroconvulsive therapy, Major depressive disorder, Delayed response, Standard treatment regime

## Abstract

**Background:**

Major depressive disorder and associated mood syndromes are amongst the most common psychiatric disorders. To date, electroconvulsive therapy (ECT) is considered the most effective short-term treatment for patients with severe or treatment-resistant depression. In clinical practice, there is considerable variation in the ECT dosing schedule, with the number of sessions typically ranging from 6 to 12, with early antidepressant effects being predictive of increased positive outcomes. We describe here an unusual case of a female patient with severe depression who did not respond to ECT until the 11th session, after which she had shown a drastic improvement in her mental state.

**Case presentation:**

A 75-year-old female presented to the old age psychiatry inpatient unit with new onset dysphoric mood, anhedonia, and severe negativity. She scored 23 on the 17-item Hamilton Rating Scale for Depression (HAM-D), and was rated 6 on Clinical Global Impression severity (CGIS) by the responsible clinician. She suffered from post-natal depression fifty years ago and was successfully treated with ECT. She was therefore initiated on a course of ECT treatment. Her condition initially deteriorated, displaying features of catatonia and psychosis, unresponsive to ECT treatment or concurrent psychotropic medications. After 11th ECT session, she started to show signs of clinical improvement and returned close to her baseline mental state after a total of 17 ECT sessions. She remained well 3 months post-treatment, scoring 4 on HAM-D, Clinical Global Improvement or change (CGI-C) rated as 1 (very much improved). The diagnosis was ICD-10 F32.3 severe depressive episode with psychotic symptoms.

**Conclusions:**

we describe here an unusual case of delayed response to electroconvulsive therapy in the treatment of severe depressive disorder. Studies have shown the number of acute ECT treatments to be highly variable, affected by a number of factors including treatment frequency, condition treated and its severity, the ECT technical parameters, as well as concurrent use of pharmacological treatment. This may call for re-consideration of the current ECT treatment guidelines, requiring more research to help stratify and standardize the treatment regime.

## Background

Major depressive disorder (MDD) and associated mood syndromes are amongst the most common psychiatric disorders. MDD can at times become debilitating, or at worst, life-threatening. In general, antidepressant medications can be effective in treating MDD, but they fail to achieve remission in approximately 1 in 3 patients [1].

Electroconvulsive therapy (ECT) is considered the most effective short-term treatment for patients with severe or treatment-resistant depression, with 70–90% of patients showing improvement [2]. ECT involves the application of electricity to the scalp in order to induce seizure activity [3].

There is a considerable variation in ECT dosing schedules in clinical practice. During an acute course of the treatment, ECT is given as a twice-weekly regime in the United Kingdom [4], and twice or thrice-weekly as recommended in the United States [5]. More frequent schedule may be given in situations where rapid onset of response is of prime importance. The number of sessions typically range from 6 to 12 [4]. It has been suggested that individuals with schizophrenia may need 12 to 20 sessions [6]. Rarely, some patients may be prescribed ECT as ‘continuation’ (C-ECT) or ‘maintenance’ ECT (M-ECT) to prevent relapse and recurrence, respectively [7].

The ECT handbook of the Royal College of Psychiatrists stated that clinicians may wish to reassess the need for ECT if there was no response after six sessions. If there is no response within 12 treatment sessions, it is unlikely to have a ‘sustained response to ECT [8].’

The number of ECT sessions required to elicit improvement tends to vary depending on the intensity of ECT, where a thrice weekly regime yields faster response than twice weekly [9]. Other factors associated with a rapid response include the severity of depression and seizure Energy Index [10, 11]. The pulse width of the ECT stimulation and electrode placement have also been implicated in the speed of response [12, 13]. A few of the important factors affecting ECT efficacy are summarised in Table 1. One study had concluded that the average number of sessions required to elicit remission in depression was 10.9 (± S.D. 4.3) [14]. Another showed a 50% response rate to ECT after the 12th session [15], whilst some patients showed response to ECT after the first session [16]. To date, we are not aware that there is robust clinical evidence to support using 12 sessions as a cut-off point, apart from the legal framework of ECT. Thirtalli et al. reviewed the factors influencing the number of ECT sessions, from which no recommendations could be drawn, and the number of ECT sessions was not included as primary outcomes in the reviewed studies [17].

**Table 1:** Factors associated with ECT efficacy

Factors	Associations	Ref.
ECT intensity	Thrice weekly ECT yields faster response than twice weekly, but induced more severe memory impairment	[9]
Severity of depression	The severity of depression predicts rapid response	[10, 11]
Presence of psychotic features	The presence of psychotic features predicts ECT remission	[11]
Seizure energy index (SEI)	High SEI is associated with a rapid response	[10]
Age	Older age predicts faster speed of response	[11, 12]
Pulse width	Brief pulse ECT (pulse width 1.0 ms) is associated with higher remission rates than ultrabrief pulse (pulse width 0.3 ms)	[12]
Electrode placement	Bilateral electrode placement is more efficient than alternative electrode placement	[13]

One of the variables predictive of increased positive outcomes is the early antidepressant effects of ECT. A study by the Consortium for Research in ECT has indicated that more than half of patients treated with ECT showed improvement after 3 sessions, and 65% achieved remission after 10 sessions [16]. Likewise, Tsuchiyama et al. 2005 reported that response by session 3 of ECT treatment predicted long-term efficacy in relieving depression [18]. On the contrary, a study examining the effectiveness of ECT in adolescents suggested that early lack of response does not necessarily predict a lack of response at the end of the ECT course, with significant clinical improvement seen after 12 sessions [19].

In this article, we outline a case of a female patient, who presented with severe depressive episode. She initially failed to respond to her ECT treatment, until the 11th session, when she had a drastic improvement in her clinical presentation.

## Case presentation

A 75-year-old female patient presented to the old age psychiatry inpatient unit with a 10-week history of deteriorating depressive symptoms, triggered by a telephone scam. She had repeatedly attempted to slit her wrists with a knife, and developed concerns about having cancer following two brief illnesses with infections. On admission, she presented with dysphoric mood, anhedonia, and severe negativity. She described herself as not having ‘any thoughts in my head,’ and ‘frightened.’ She showed evidence of catastrophizing, and appeared hypervigilant. Her food and fluid intake had been poor over the past 10 weeks.

In the past, she suffered from post-natal depression fifty years ago and was successfully treated with ECT. In the intervening years, she did not suffer from any episodes of severe mental illness, with no depression or mania. Physically, she suffered from hypertension, type 2 diabetes mellitus, chronic obstructive pulmonary disease, and polymyalgia rheumatica for which she was on a reducing dose regime of prednisolone (4 mg OD on admission, reduced by 1 mg every 12 week). She was a non-smoker, and there was no history of alcohol or substance misuse. She lives with her husband and has two supportive children living nearby.

On examining her mental state, she was catatonic, displaying excessive motion intermittently including bilateral non-Parkinsonian motion of the upper limbs and lip licking. Her speech was slow and interrupted in flow. She felt low in mood and was negative in her outlook. She was not formally thought disordered, and she was cognitively intact. She had insight into the deterioration of her mental state and agreed to informal admission to the hospital for assessment and treatment.

Admission blood tests were normal. She was not anaemic, thyroid function tests were normal.

Initially on the ward, she appeared settled and was able to hold conversations with others. She was periodically anxious, requiring encouragement with food and drink intake. Due to her good response previously, the treatment team arranged a course of ECT during the admission, and she was deemed to have capacity to consent to the treatment. Prior to starting ECT, a 17-item Hamilton Rating Scale for Depression (HAM-D) was obtained, for which she scored 23, indicative of severe depression. A Mini-mental state examination (MMSE) was performed, when converted into Addenbrooke’s Cognitive Examination III (ACE-III), gives 69–73/100. Clinical Global Impression Severity (CGIS) by the responsible clinician was rated 6 (severely ill).

**Fig. 1:**
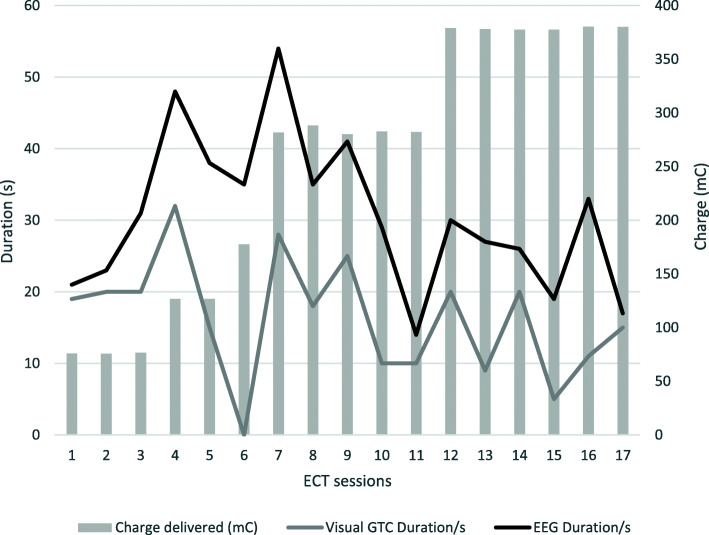
ECT session visual and EEG seizure duration in s, charge delivered in mC

ECT was performed with Thymatron® System IV, using bitemporal electrode placement with a pulse width of 0.50 millisecond. The details of each ECT session are summarised in Table 2 and Fig. 1.

**Table 2:** Details of ECT administration

ECT session	BL/UL	Mental Capacity	Dose^a^	Charge delivered (mC)	Current (A)	Frequency (Hertz)	Pulse width/ms	Stimulus duration/s	Seizure duration		Anaesthesia	Other information
									Visual GTCS/s	EEG/s		
1	BL	Consented	10% did not elicit adequate seizure. Increased to 15%	75.9	0.90	20	0.50	4.2	19	21	Propofol 90 mg Suxamethonium 40 mg	800 ml Hartmann’s solution IV given
2	BL	Section 62	15%	75.7	0.90	20	0.50	4.2	20	23	Propofol 70 mg Suxamethonium 35 mg	Unable to consent. Given under section 62 1 L Hartmann’s solution IV given
3	BL	Section 62	15%	76.6	0.91	20	0.50	4.2	20	31	Propofol 80 mg Suxamethonium 40 mg	1 L Hartmann’s solution IV given
4	BL	T6	25%	126.6	0.90	20	0.50	7.0	32	48	Propofol 70 mg Suxamethonium 35 mg	800 ml Hartmann’s solution IV given
5	BL	T6	25%	126.7	0.91	20	0.50	7.0	15	38	Propofol 70 mg Suxamethonium 35 mg	1 L Hartmann’s solution IV given
6	BL	T6	25% did not elicit visual seizure. Increased to 35%	177.7	0.91	30	0.50	6.5	Not observed	35	Propofol 90 mg Suxamethonium 35 mg	Unable to obtain MMSE due to severe catatonia. 500 ml Hartmann’s solution IV given
7	BL	T6	Increased to 55%, as no visual seizure in session 6	281.7	0.91	40	0.50	7.7	28	54	Propofol 70 mg Suxamethonium 40 mg	Difficult to engage prior to treatment due to distress and agitation 500 ml Hartmann’s solution IV given
8	BL	T6	55%	288.2	0.94	40	0.50	7.7	18	35	Propofol 80 mg Suxamethonium 35 mg	Unable to obtain MMSE due to severe catatonia 500 ml Hartmann’s solution IV given
9	BL	T6	55%	280.3	0.91	40	0.50	7.7	25	41	Propofol 80 mg Suxamethonium 40 mg	
10	BL	T6	55%	282.7	0.92	40	0.50	7.7	10	29	Propofol 80 mg Suxamethonium 35 mg	
11	BL	T6	55%	282.3	0.92	40	0.50	7.7	10	14	Propofol 80 mg Suxamethonium 40 mg	500 ml IV 0.9% saline given
12	BL	T6	75%	379.0	0.90	60	0.50	7.0	20	30	Propofol 80 mg Suxamethonium 40 mg	
13	BL	T6	75%	378.0	0.90	60	0.50	7.0	9	27	Propofol 80 mg Suxamethonium 40 mg	
14	BL	T6	75%	377.6	0.90	60	0.50	7.0	20	26	Propofol 80 mg Suxamethonium 40 mg	
15	BL	T6	75%	377.6	0.90	60	0.50	7.0	5	19	Propofol 80 mg Suxamethonium 40 mg	
16	BL	T6	75%	380.4	0.91	60	0.50	7.0	11	33	Propofol 100 mg Suxamethonium 40 mg	
17	BL	T6	75%	380.2	0.91	60	0.50	7.0	15	17	Propofol 100 mg Suxamethonium 40 mg	
18								Cancelled due to pyrexia of 37.7 C° pre-ECT. COVID-19 was suspected^b^.				

^a^ECT dose at which a seizure response is visualised

^b^2019-nCoV RNA Not Detected

*BL/UL* bilateral/unilateral temporal electrode placement, *GTCS* generalized tonic-clonic seizure, *EEG* electroencephalogram

Unfortunately, whilst on the ward, her clinical condition deteriorated. Her catatonia worsened considerably. She displayed virtually no interaction with external world. She showed mutism, fixed, non-reactive gaze. At times she would whisper ‘locked in,’ ‘I can’t’. She repeatedly exhibited a rocking motion of her torso with mild rigidity of her limbs. She was unable to be encouraged off her armchair and was unable to walk. She was unable to eat and drink, and was often unable to take oral medications. Prior to the second planned treatment, the team felt that she lacked capacity to consent to further sessions.

A Mental Health Act Assessment was arranged, and she was detained under Mental Health Act Section 2. Urgent ECT was prescribed under section 62, whilst a second opinion appointed doctor (SOAD) was sought for to grant her further ECT sessions. A total of 12 ECT sessions were granted.

After eight further sessions, the patient’s mental state did not improve. She continued to display minimal engagement. She often refused her medications, and her food and fluid intake was minimal. As a result, she underwent intravenous fluid therapy several times due to dehydration causing a deterioration of renal function. Treatment was given under Mental Health Act 1983 (amended 2007).

After 4th and 8th ECT sessions, repeated attempts were made by the ECT doctor to perform a MMSE, but she was deemed too unresponsive to answer the questions.

At the same time, several psychotropic medications were trialled. The date of their commencement and dose changes in relation to her ECT treatment are summarised in Table 3.

**Table 3:** Timing of medication changes in relation to ECT sessions over the admission period

Day of stay	ECT Session	Medication Changes
D1		Sertraline increased to 150 mg OD
D4	Session 1	
D11	Session 2	
D14	Session 3	
D17		Sertraline increased to 200 mg OD
D18	Session 4	
D22	Session 5	
D24		Quetiapine 50 mg BD
D25	Session 6	
D28	Session 7	
D31		Quetiapine increased to 75 mg BD
D32	Session 8	
D34	Session 9	
D35		Quetiapine increased to 100 mg BD
D39	Session 10	Lamotrigine 25 mg OD started
D40		Quetiapine stopped. Olanzapine 5 mg OD started
D42	Session 11	
D45		Olanzapine increased to 7.5 mg OD
D46	Session 12	
D49	Session 13	
D53	Session 14	Lamotrigine increased to 50 mg OD
D56	Session 15	
D60	Session 16	Lamotrigine increased to 75 mg OD
D63	Session 17	
D67		Lamotrigine to 100 mg OD

As a result of her lack of response to treatment, her diagnosis was reviewed and affirmed by the treatment team. Six further ECT sessions were granted through a Second Opinion Appointed Doctor (SOAD).

After 11 ECT sessions, she started to show signs of improvement. She started to walk around the ward and engage in various group activities. She started to accept her medications with less prompting. Her food and drink intake improved significantly, and she could eat independently. Her engagement with staff gradually improved, from making appropriate facial expressions to starting verbal communication. By the end of her ECT course, she could spontaneously engage in conversations and reported to us that ‘I am back.’ She was able to reflect on how severely unwell she was and felt ready to be discharged home. In view of her clinical improvement, ECT treatment was stopped. Extracts of clinical documentation on her progress have been summarized in Table 4.

**Table 4:** Patient progress on the ward as documented in the electronic note system

ECT Session	Prior to ECT	Post ECT in recovery	Progress on the ward
1	Settled, anxious at times, joined in with activities. Able to hold a conversation Consented to ECT. Quiet on arrival, but did answer 4/5 orientation questions correctly	Anxious, quiet, but pleasant on approach. Compliant with medications	Reported to be highly anxious, kept saying ‘no’ or ‘I don’t know’ – unable to give capacitous consent. Moderate evidence of psychomotor retardation and evolving mutism. Did not appear to be responding to unseen external stimuli. She required full support for her personal care
2	Unable to consent	On return to the ward, she refused medications, spitted them out and had her mouth closed	Poor oral and fluid intake, received IV fluid treatment due to dehydration and deteriorating renal function
3			
4	Very anxious and unable to answer any of the orientation questions	Able to drink full cup of squash and ate 2 biscuits. Completed 4/5 orientation questions	Upon returning to the ward, she became very anxious and hardly communicative. She was visibly shaking and humming
5	Very anxious and unable to answer any of the orientation questions		Intermittent compliance with prescribed oral medication
6	Remained anxious and reluctant. Unable to answer any questions	Had half a beaker of squash and IV Hartmann’s. Unable to obtain MMSE	Minimally interactive, and will respond with single words (no, no, no)
7	Had to be brought on a trolley due to high level of anxiety, distress and agitation	Had some tea and biscuits – couldn’t answer orientation questions afterwards	She was having minimal conversation and continued to say ‘no, no, no.’
8	Very anxious and was reluctant. Unable to answer any of the orientation questions	Had a mug of tea 280mls as well as 500 ml of Hartmann’s Fluid IV	She has been keeping tablets in her mouth at times. Staff had to escort her to the toilet using a wheelchair
9		After the session, had tea, toast and biscuit. Conversed with staff about dieting, exercise and dogs	She returned to the ward with a smile. Then in the afternoon, she returned to the anxious state. She did manage all her meal
10	She had been variable in presentation. When she is less anxious, she could feed herself. Other times, she refused to eat and drink	She had been sat in the communal area post-ECT.	She was reasonably settled but gradually got anxious and had been repetitive and making sounds
11	SOAD applied for 6 further ECT sessions	She was very calm, however did not interact with staff other than with short answers. She accepted a mug of tea and one and half slices of toast and marmalade. She was unable to answer any of the orientation questions	She has been out in the garden for a walk. She joined in the colouring group and participated well. Accepted medication with less prompting
12		After the treatment, she was much calmer, however appeared confused at times. She had tea, cheddars and toast	She engaged well in physiotherapy strength and balance sessions
13		Calm. Did not answer any of the orientation questions. The only thing she said was ‘what is my name.’ She walked calmly back to the ward with staff	Eating and drinking well. Taking food in the canteen now, despite eating slowly. Minimal assistance required with personal care
14		Accepted tea and toast. She was able to answer 2/5 of the orientation questions, however did say ‘my name used to be …’	She had been settled and calm, spent time in communal area with others. She engaged well when spoken to and has not been making any noise.
15		She was able to tell us her name, date of birth, however followed each answer by ‘well it used to be’	When staff talked to her, she would always respond back with talking to staff. At times, she did smile to staff
16	She was able to walk to the clinic with a member of staff and answered 3/5 of the orientation questions. She became increasingly anxious when being prepared for ECT, however remained cooperative throughout	She was able to tell us her name and date of birth, but responded ‘I have no idea’ when asked where she currently is	Walked back to the ward with no concerns
17	She was able to answer 3/5 of the orientation questions	She had cheddars and tea after the treatment. She completed 4/5 orientation questions and initiated the conversation briefly	She joined drama therapy group for the first time. Although she appeared quiet throughout the session, she contributed vocally and artistically to group exercises
18	ECT cancelled due to temperature of 37.7 °C		She was bright in mood, smiling more, playing dominoes with other service users. Eating and drinking well. She could reflect on how severely unwell she was

The diagnosis was made during the admission – ICD-10 F32.3 severe depressive episode with psychotic symptoms [20]. She showed features of psychosis during the admission period. She frequently appeared guarded and suspicious, at time frightened. She at times reported that ‘people on the ward were not eating or drinking,’

Three months post-treatment, she scored 4 on HAM-D, and scored 89–93/100 for ACE-III (converted from MMSE). Clinical Global Improvement or change (CGI-C) was rated 1 (very much improved). Efficacy index was rated at 02 (vast improvement with side effects that do not significantly interfere with patient’s functioning).

## Discussion and conclusion

We describe here a patient with a diagnosis of severe depression. She did not respond to electroconvulsive therapy until the 11th session, after which she had shown a drastic improvement in her mental state. The case is considered unusual. In the literature, the number of sessions during an acute course of ECT typically ranges from 6 to 12, usually given twice a week [4]. Early antidepressant effects of ECT is predictable of increased positive outcomes (6–7). Rarely, individuals with schizophrenia may require 12 to 20 sessions to elicit a response to treatment [5].

Compared to pharmacological treatment, ECT remains the most effective short-term treatment for patients with severe or treatment-resistant depression, with 70–90% of patients showing improvement [2]. In this case, a longer course of ECT has likely contributed to the patient’s response and remission.

There may be some debate surrounding the initiation of the ECT course in this patient. The empirical titration method aims to establish seizure threshold in the first session (titration session), and from session two onwards, therapeutic sessions are given with the stimulus at 1.5 times of the seizure threshold [8]. However, there has been questions surrounding the current threshold titration method of being too uncertain for valid optimisation or individualization of dose [21]. In this patient, the dose was not up-titrated, given the adequate seizure duration, good quality EEG fit during session one, balanced with potential cognitive side effect in this 75-year-old patient.

Other factors that might have contributed towards the improvement in this patient’s mental state includes a change in her medication regime. By the 10th session, she was taking three adjunctive pharmacological treatments including quetiapine, olanzapine, and lamotrigine. Quetiapine has demonstrated up to 48% response rate in combination with SSRIs and have been approved for adjunctive treatment of MDD by the Food and Drug Administration (FDA) [22]. Olanzapine, when combined specifically with fluoxetine, demonstrated 60% response amongst patients with treatment-resistant depression [23]. Lamotrigine is an anti-convulsive treatment that is currently licensed as an adjunctive therapy of bipolar disorder [24]. However, it was introduced by the treatment team following a lack of clinical response from the other agents and concurrent ECT treatment. Several studies have shown clinical improvement after augmentation with lamotrigine in treatment-resistant unipolar depression [25, 26].

The anti-epileptic effect of lamotrigine can theoretically inhibit the efficacy of ECT in inducing seizure activity. However, case reports/series have shown minimal or no influence on seizure and/or seizure duration [23]. In this patient, there was an increase in seizure threshold at session 12, with the introduction of lamotrigine being one possible contributing factor. However, it more than likely reflected the natural course of an ECT treatment regime. In addition, sertraline and olanzapine can theoretically lower seizure threshold, and some data have suggested beneficial and additive efficacy of second-generation antipsychotics (SGAs) to ECT, notably with clozapine [27].

The ECT handbook of the Royal College of Psychiatrists stated that clinicians may wish to reassess the need for ECT if there is no response after six sessions. If there is no response within 12 treatment sessions, the patient is unlikely to have a ‘sustained response to ECT’ [8]. This patient displayed a delayed response, which is an exception to the typical response pattern to ECT treatment for major depressive disorder. However, studies have shown the number of acute ECT treatments to be highly variable, and its efficacy is affected by a number of factors including treatment frequency [9], condition treated and its severity [10], the ECT pulse width [12] and electrode placement [13]. The concurrent use of pharmacological treatment for psychiatric and non-psychiatric conditions can also interact with ECT treatment [27]. This may call for a re-consideration of the ECT treatment regime, especially in cases where response may not have been observed by the later part of a treatment course. Moreover, further research is needed to help stratify and standardize the ECT treatment regime.

## Data Availability

All data generated or analysed during this study are included in this published article.

## Abbreviations

ECT: Electroconvulsive therapy
HAM-D: Hamilton Rating Scale for Depression
CGIS: Clinical Global Impression severity
CGI-C: Clinical Global Improvement or Change
ICD-10: International Classification of Disease 10th revision
MDD: Major depressive disorder
MMSE: Mini-mental state examination
ACE-III: Addenbrooke’s Cognitive Examination III
SOAD: Second opinion appointed doctor
SGA: Second-generation anti-psychotics
